# From Classical Radiation to Modern Radiation: Past, Present, and Future of Radiation Mutation Breeding

**DOI:** 10.3389/fpubh.2021.768071

**Published:** 2021-12-21

**Authors:** Liqiu Ma, Fuquan Kong, Kai Sun, Ting Wang, Tao Guo

**Affiliations:** ^1^Department of Nuclear Physics, China Institute of Atomic Energy, Beijing, China; ^2^National Innovation Center of Radiation Application, Beijing, China; ^3^National Engineering Research Center of Plant Space Breeding, South China Agricultural University, Guangdong, China; ^4^Key Laboratory of High Magnetic Field and Ion Beam Physical Biology, Hefei Institutes of Physical Science, Chinese Academy of Sciences, Hefei, China

**Keywords:** mutation breeding, classical radiation, particle radiation, space radiation, mutagenesis

## Abstract

Radiation mutation breeding has been used for nearly 100 years and has successfully improved crops by increasing genetic variation. Global food production is facing a series of challenges, such as rapid population growth, environmental pollution and climate change. How to feed the world's enormous human population poses great challenges to breeders. Although advanced technologies, such as gene editing, have provided effective ways to breed varieties, by editing a single or multiple specific target genes, enhancing germplasm diversity through mutation is still indispensable in modern and classical radiation breeding because it is more likely to produce random mutations in the whole genome. In this short review, the current status of classical radiation, accelerated particle and space radiation mutation breeding is discussed, and the molecular mechanisms of radiation-induced mutation are demonstrated. This review also looks into the future development of radiation mutation breeding, hoping to deepen our understanding and provide new vitality for the further development of radiation mutation breeding.

## Introduction

Crops provide the most basic guarantee for human survival on Earth, its domestication plays an important role in developing wild plants to produce cultivated crops through the long-term screening of desirable characteristics caused by gene mutations (1, 2). However, spontaneous mutation appears at an extremely low frequency in nature (~10^−6^), rendering the process of excellent variety cultivation screening tedious. How to accelerate the frequency of mutation has always been a key problem in crop variety development, with a long history from natural evolution to cross breeding and mutation breeding in crop breeding.

Mutation breeding refers to the method of using artificial mutagenesis to obtain new biological cultivars, mainly through chemical or radiation mutagenesis. Chemical mutagenesis refers to the biochemical reaction between chemical agents and genetic material, and the result is mostly point mutations in genes. Although chemical mutagenesis is effective, its environmental optimization and biological safety need to be improved. Comparatively, radiation mutagenesis has the characteristics of more complex genetic mutations and more beneficial mutant phenotypes.

Radiation mutation breeding is generally divided into classical radiation mutation breeding, particle mutation breeding and space radiation mutation breeding. Classical radiation mutation breeding methods mainly include X-ray and gamma ray applications. As a commonly used method, classical radiation mutation breeding has been proven to be useful for crop variation, which mainly refers to the process of using various rays to induce a large number of genomic mutations and speed up the production of mutant traits through energy deposition directly or indirectly onto DNA. This approach offers the possibility of inducing desirable attributes that either cannot be expressed in nature or have been lost during evolution, and a large number of new varieties widely used in production have been bred by classical radiation mutation technology (3).

Particle mutation breeding mainly uses accelerated particles, such as heavy-ions or protons. They have unique physical properties, such as diversified radiation parameters, complex track structure and depth-dose distribution. Accelerated particle has been considered a powerful mutagen for crop breeding because it induces excellent biological mutagenic effectiveness at relatively low radiation doses (4). A notable feature of the particle radiation mutagenesis technology is that it can produce novel cultivars with good traits without affecting other phenotypes (5).

With the steady advancement of manned space projects, space exploration activities will become more frequent in the future. The space environment refers to the outer space outside the atmosphere accompanied by radiation, microgravity, and alternating magnetic fields. This special and complex environment brings new opportunities for mutation breeding. Compared with traditional radiation, space breeding has the characteristics of a high mutation frequency and multiple directions; its mutation rate can reach 10% (6), and a series of new plant varieties have been developed in this way (7, 8).

Radiation mutation breeding has played an important role in the cultivation of new crop varieties. In this review, we first briefly discuss achievements through radiation breeding in recent decades as well as some concerns on the process and mechanism of classical radiation, accelerated particle and space radiation mutagenesis. This review will deepen our knowledge and provide a theoretical foundation for improving the efficiency of future crop radiation mutation breeding and promoting improvement under the challenge of other newly emerging breeding methods.

## Development And Mechanism Of Classical Radiation Mutation Breeding

### Past and Present of Classical Radiation Mutation Breeding

**Figure 1:**
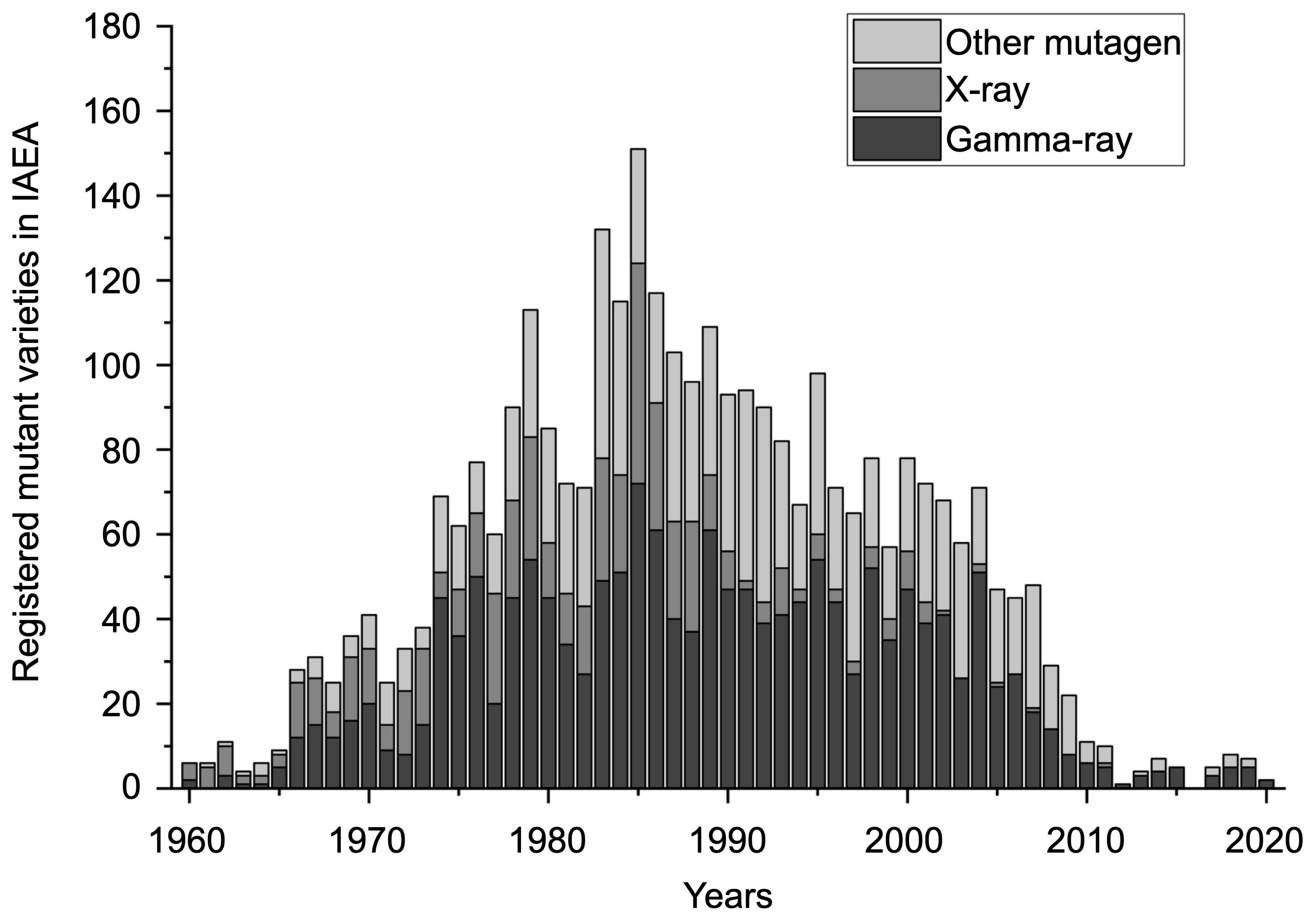
Numbers of mutant varieties registered in IAEA during 1960–2020 (data from IAEA Mutant Variety Database).

Radiation was suggested as a mutagen since Muller demonstrated that exposure to X-rays can cause genetic mutations in 1928 (9). After Stadler first published papers on mutations induced by irradiation in maize and barley (10, 11), radiation has been widely applied to develop new cultivars used for crop production and as genetic resources. Compared to other breeding methods, such as cross-breeding and chemical mutagenesis, radiation mutation breeding has incomparable advantages, with a wide mutation spectrum and high mutation efficiency (12). To date, 3,365 mutant varieties have been registered in the Mutant Variety Database of the International Atomic Energy Agency (IAEA), and more than 1,000 new varieties have been used and promoted worldwide. Here, we analyse the varieties bred by mutation in the past 60 years from 1960 to 2020 (13). Figure 1 shows that most of the registered varieties bred by various mutation approaches were concentrated before 2010, with a peak in the 1980s. Seventy percentage of the overall registered varieties were produced by classical gamma rays and X-rays irradiation, which laid a crucial foundation for the agriculture development. However, great challenges have been brought to traditional breeding methods with the development of advanced mutation technology, such as targeted gene editing represented by CRISPR (clustered regularly interspaced short palindromic repeat) technology in recent years (14), which might explain the steep decline in the number of varieties registered in Mutant Variety Database of IAEA after 2010. Of course, it is possible that many new varieties might have been bred by traditional radiation during this period without being registered. Nevertheless, the downward trend suggests that after years of continuous breeding, variation traits have been basically saturated from a macroscopic view, especially in some important crop varieties, and it is difficult to obtain new breakthrough variation traits under current knowledge on radiation mutation. Therefore, more research should be performed to elucidate the mechanism of radiation mutagenesis.

### The Mutagenesis Mechanism Under Classical Radiation Mutation Breeding

#### Radiation-Induced DNA Damage

**Figure 2:**
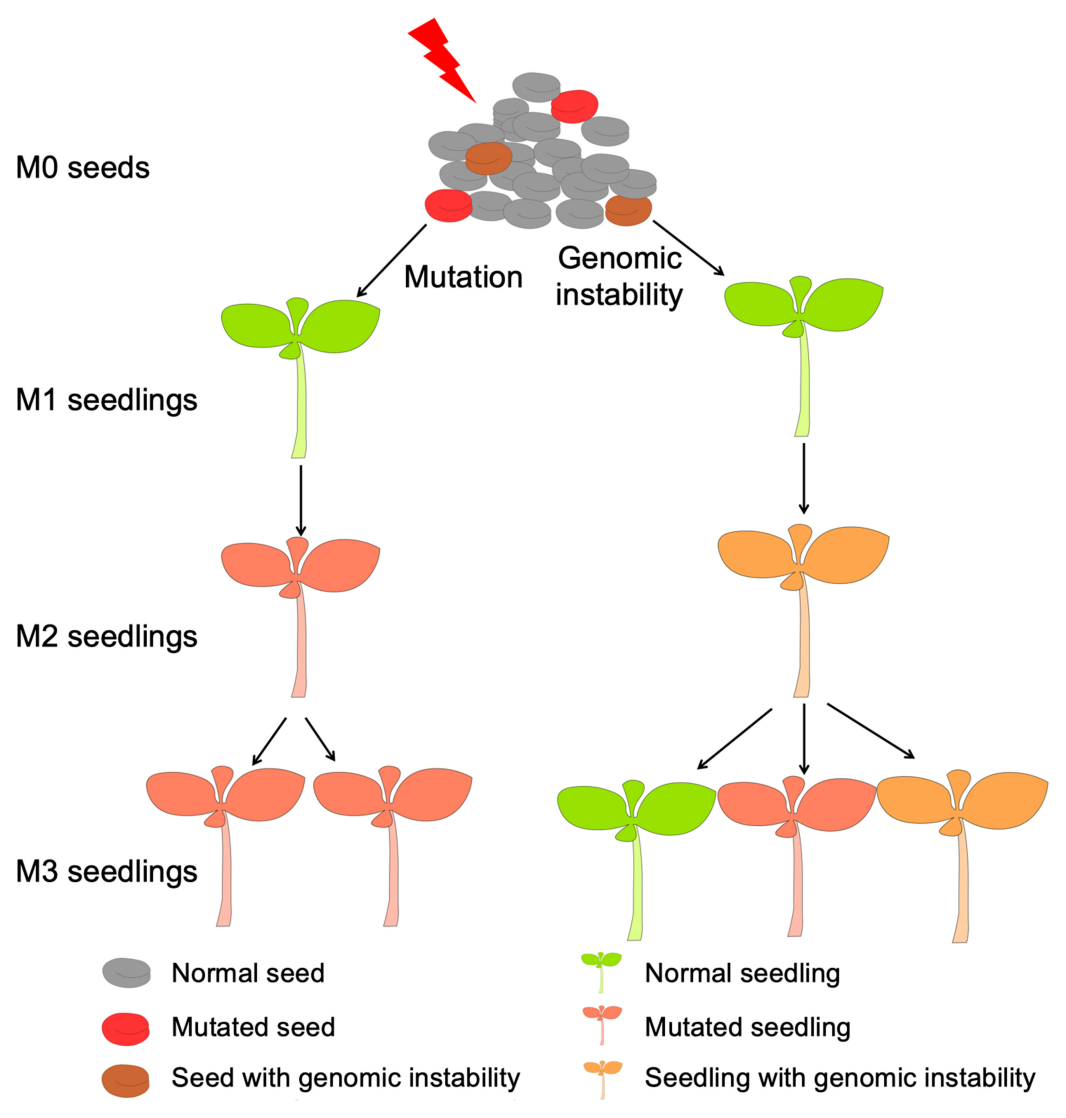
Two sources of the mutant in progeny, left shows that the mutant originated from direct transmission from irradiated seeds, right shows that the mutant was formed through genomic instability.

The process of radiation mutation breeding begins with interactions between radiation and DNA, including direct structural and functional changes to DNA molecules via radiation energy and indirect damage by free radicals generated through interactions between water molecules and ionizing radiation (15). To maintain genomic integrity, cells have evolved a set of repair mechanisms to address DNA damage. Indeed, the repair method is invoked according to the type of DNA damage incurred (16). DNA damage can be divided into two categories: single-strand break (SSB) and double-strand break (DSB). The SSB repair pathways are mainly base excision repair (BER), nucleotide excision repair (NER) and mismatch repair (MMR). In contrast, DSBs are mainly repaired by non-homologous end-joining (NHEJ) and homologous recombination (HR) (16, 17). However, DNA damage is not equivalent to mutation. If DNA damage is repaired correctly, no mutation will remain. Gene mutation is the result of “errors” in the process of DNA damage repair. Some of these errors are accidental, such as replication errors caused by some single-strand breaks not being detected before DNA replication, unstable DNA single strands in the process of repair, and the participation of low-fidelity polymerase, among others; the mutation type is basically a point mutation with base substitution (18). For severe DSBs, deletion and translocation of fragments are introduced in the repair process (19). If these mutations are retained in subsequent cell division and inherited by progeny, they become the source of mutant traits, as shown in Figure 2.

#### Radiation-Induced Genomic Instability

In addition to the direct inheritance of DNA damage caused by radiation to progeny, there is another method of inheritance that can result in mutant traits in offspring, as shown in Figure 2. As mentioned above, the genetic stability of the genome is key to maintaining normal cell proliferation and differentiation. Normal cells have efficient DNA damage monitoring and response mechanisms to deal with the pressure on the genome caused by internal and external stress, and maintain genome damage and repair in a relatively balanced state. When this balance is broken, however, cells enter a DNA mutation susceptibility state called genomic instability, which can be caused by genetic mutation or epigenetic modification (20). Radiation-induced genomic instability is a concept that describes delayed and persistent genetic alterations in progeny of the irradiated cells, which was first detected in *in vitro* cell system experiments in the 1950s (21). Subsequent studies have found that gamma rays, neutrons, protons and α particles can induce genomic instability in cells, which manifests as an increase in various types of mutations, such as single-nucleotide mutations, an increase or decrease in genomic copy number, gene amplification, rearrangement and deletion (22, 23). Using a homologous recombination reporter system, radiation-induced genomic instability has also been confirmed in plant systems, with increased frequencies of homologous recombination persisting in subsequent generations (24–26).

## Development And Mechanism Of Particle Radiation Mutation Breeding

### The New Generation Particle Radiation Mutagenesis Technology

Unlike classical gamma rays and X-rays, which are essentially electromagnetic waves, the emerging mutagens represented by accelerated heavy-ions or protons are essentially charged particles. Compared with classical radiation, accelerated particle irradiation can deposit more energy along the ion track and can maintain a higher mutation frequency and mutation spectrum at a relatively low dose (4). This is because accelerated particles with high linear energy transfer (LET) cause high-density ionization along the ion track, causing a large amount of damage to DNA in a small area, which is termed clustered DNA damage (27–29). Such clustered DNA damage is difficult to repair effectively and correctly, leading to the generation of free DNA fragments, which contribute to the formation of chromosome rearrangements and large deletions (30–32). These rearrangements and large deletions can generate more combinations of gene mutation sites, thereby breaking the linkage inheritance of traits, and it is expected that more mutants with excellent traits will be obtained.

The technology of particle radiation mutagenesis based on advanced particle accelerators originated in Japan in the 1990s (4). Although there are many particle accelerator facilities in the world, most of them are used for nuclear physics research, and there are few irradiation facilities that can be used for crop breeding. Particle accelerators can be divided into medium- and high-energy (MeV or GeV level) particle accelerators and low-energy (KeV level) particle accelerators according to the energy of the accelerated particles. In general, medium- and high-energy particles are considered to penetrate the target material, whereas low-energy particle cannot penetrate the target material, which is commonly referred to as ion implantation. To date, the medium- and high-energy particle accelerator facilities used for particle radiation mutation breeding include RIBF of the Institute of Physical and Chemical Research (RIKEN, Japan), TIARA of the National Institutes for Quantum Science and Technology (QST, Japan), W-MAST of the Wakasa Wan Energy Research Center (WERC, Japan), LNS of the National Institute for Nuclear Physics (INFN, Italy), HIRFL of the Institute of Modern Physics, Chinese Academy of Sciences (CAS-IMP, China), and CYCIAE100 of the Chinese Institute of Atomic Energy (CIAE, China) (Table 1). In the field of low-energy particle mutagenesis, the most representative research facilities are the IBBe-Device of the Hefei Institute of Physical Science, Chinese Academy of Sciences (CAS-HIPS, China), IBBT of Chiang Mai University (CMU, Thailand)..

**Table 1:** Particle accelerator facilities that can be used for radiation breeding.

**Medium- and High-energy facility**	**Institute**	**Ion species**	**Energy (MeV)**	**LET (keV/μm)**	**Range in water (mm)**
RIBF	RIKEN, Japan	C, N, Ne, Ar, Fe	1,620–5,040	23–640	4–40
TIARA	QST, Japan	He, C, Ne	100–350	9–441	6–16
W-MAST	WERC, Japan	H, C	200~500.4	0.5–52	5–256
LNS	INFN, Italy	C	960	31	17
HIRFL	CAS-IMP, China	C, Ar	960–2,760	31–327	5–17
CYCIAE100	CIAE, China	H	100	0.7	76

In the early stage of the development of particle radiation mutagenesis, the technology was used for the improvement of ornamental plants, and most of the new cultivars created were exported all over the world, demonstrating its excellent cultivar improvement ability (33). Since the early twenty-first century, research on the variety improvement and mutagenesis mechanism of food crops has been successively carried out (5). More than 30 years of experience in particle radiation mutagenesis shows that the frequency of new traits in crops induced by this technology is relatively high, that the mutation trait is relatively stable and that the breeding period is greatly shortened. Mutants of food crops and ornamental plants with excellent traits generated by this technology can directly launch new cultivars or as parental materials for cross-breeding, contributing to solutions for food and environmental problems. Therefore, particle radiation mutagenesis technology has broad economic benefits and important social significance, and it is a breeding method worthy of promotion.

### Application and Mutagenesis Mechanism of High-Energy Particle Mutation Breeding

High-energy particle mutation breeding has a history of nearly 30 years thus far. The earliest high-energy particle radiation mutagenesis was used to improve the phenotype of ornamental plants, including sterility and flower color and shape. Since 2002, new flower cultivars, including the new sterile cultivar verbena and new color or shape cultivars chrysanthemum, dahlia and rose, have been developed (34). High-energy particles have also been widely used in the development of agricultural products with excellent traits, such as dwarfed buckwheat, barley and pepper (34), tearless and non-pungent onion (35), lettuce with low browning characteristics (36), rice with a stay-green phenotype (37). High-energy particle radiation mutagenesis technology also plays an important role in the field of biofuels, such as the successful mutagenesis of lipid-rich *Parachlorella kessleri* (38) and *Euglena gracilis* (39).

**Figure 3:**
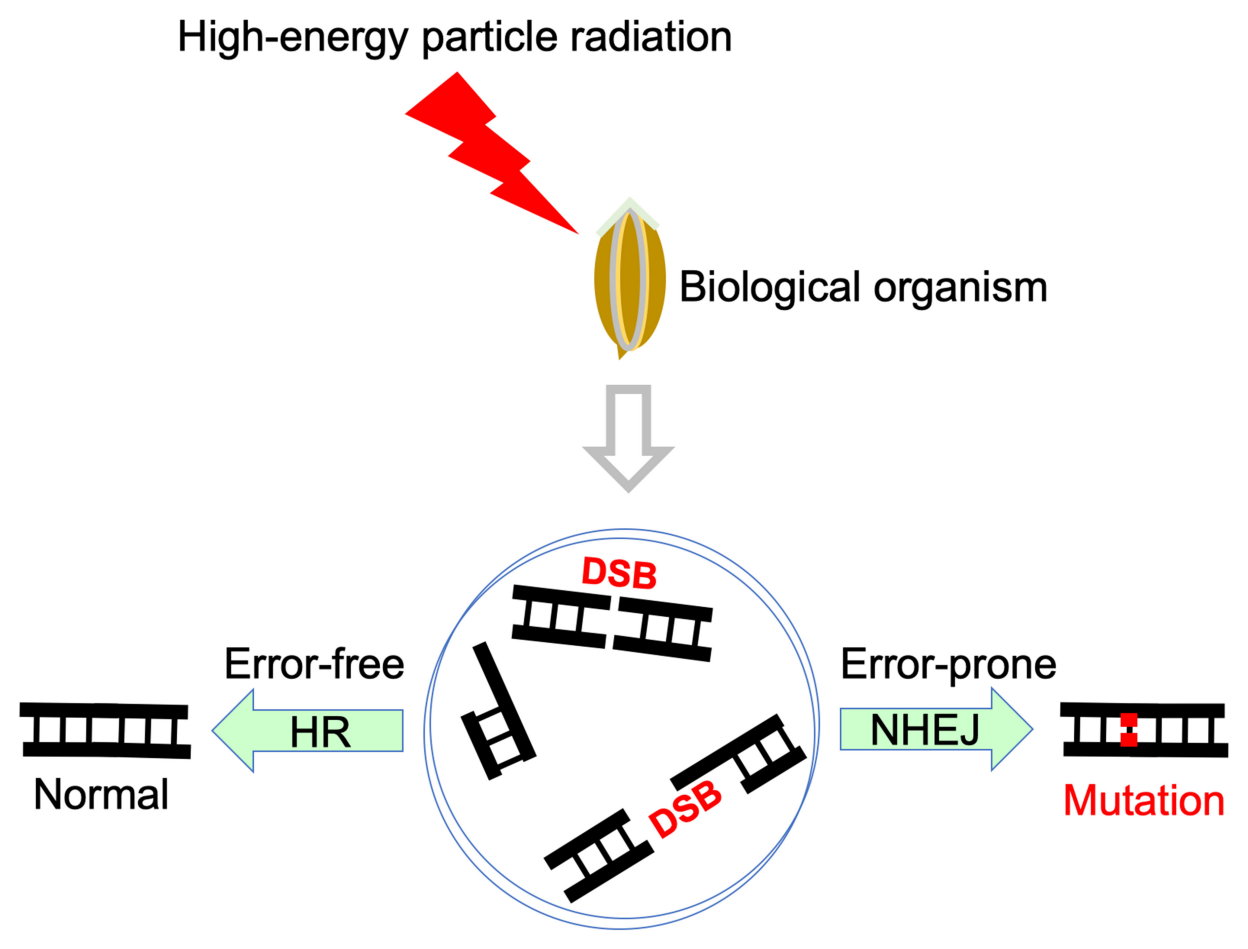
DSB repair pathways induced by high-energy particle radiation.

The successful mutagenesis of the abovementioned variants promoted the development of basic research related to particle radiation mutagenesis. To make particle radiation mutagenesis technology more efficient, it is necessary to find the most suitable physical radiation parameters, such as radiation dose and LET, which are important parameters to be considered in particle radiation mutagenesis. The survival rate of both model plants and model microbes decreases with increasing dose, and the radiation physical parameters most suitable for mutagenesis must balance survival and mutation. For example, a study by Kazama et al. using the model plant *Arabidopsis thaliana* showed that a 300–400 Gy irradiation dose and a 30 keV/μm LET carbon ion beam can generate the maximum number of mutants (40). Further mechanistic studies at the genomic level in both model plants and model microbes showed that a smaller LET is better at inducing small deletions but that larger LET radiation would lead to large deletions (41–43). In addition, through whole-genome sequencing, Kazama et al. found that relatively high LET Ar ions can cause more complicated rearrangement errors in *Arabidopsis thaliana* than C-ion irradiation technology (44). In general, mutations are generated on the basis of the damage being repaired incorrectly, and the nature of DNA damage caused by high-energy particle radiation is mainly a large number of SSBs and DSBs. SSBs are easily repaired in a short period of time, whereas DSBs constitute damage that has the greatest impact on DNA and usually requires more time to repair (45). DSB damage is mainly repaired competitively through HR or NHEJ pathways (46, 47), as shown in Figure 3. The HR is highly accurate while the NHEJ is error prone. For example, a study by Ma et al. using the model microbe *Neurospora crassa* reported that compared with the NHEJ-deficient strain, the HR-deficient strain results in higher mutation frequency after high-energy particle irradiation (48). Another study based on rice transcriptome sequencing suggested that alternative NHEJ (aNHEJ) may be involved in the DNA repair of complex damage induced by high-LET irradiation (49). These studies revealed that NHEJ has a greater contribution to mutagenesis, and NHEJ enhancement and/or HR suppression strategies may significantly increase the mutagenic efficiency of high-energy particle irradiation.

Furthermore, the new trait mutants obtained by particle radiation mutagenesis are suitable for gene function mining, gene mapping, and even the creation of elite alleles. Several mutants of *Arabidopsis thaliana*, wheat, buckwheat, and rice have been isolated following particle irradiation based on gene mutations formed through error-prone DSB repair pathways such as canonical NHEJ and aNHEJ. These mutants can help us to understand the function of affected genes; for example, FRL1 impacts sepal development (50), VRN1 influences flowering (51), S-ELF3 is associated with a dwarf phenotype (52), CSV1 is related to chloroplast development (53), and LIN1 controls rice grain length (54). In addition, the Y chromosome genes of *Silene latifolia* have been physically mapped using sex chromosome mutants induced by particle irradiation (55). A recent study showed that particle radiation has the ability to create neutral alleles at the rice S1 locus, making it possible to cross distantly related species and broadening crop breeding (56).

### Application and Mutagenesis Mechanism of Low-Energy Particle Mutation Breeding

The biological effect of particle irradiation has always been an important part of radiobiology. However, for a long time, low-energy particles (10–200 KeV) have been underestimated due to their extremely short penetration depth in matter, which leads to the hypothesis that it is impossible to induce high-level biological effects via their interaction with organisms. In the early 1980s, Yu et al. first confirmed the genetic effect of low-energy particle implantation on rice (57, 58). After years of application in breeding, low-energy particle has been proven to be a high-efficiency mutagenic source for genetic modification, leading to great achievements (59, 60) and promoting the formation of a new interdiscipline of low-energy particle biology (61). Nevertheless, the debate regarding the mutagenesis mechanism of low-energy particle implantation remains. In the 1990s, Yu et al. proposed the four-factor theory of energy absorption, mass deposition, momentum transfer and charge neutralization, whereby energetic ions are transferred into organisms to cause serious etching to cells and physical damage to biological macromolecules (61). Combining the following ion channel and soft X-ray theory provided an explanation of the physical interaction process. Then, the mutagenesis mechanism of low-energy particle implantation was further elucidated from the biological process. Considering that the theoretical range for low-energy particles in water is <1 μm, which could hardly penetrate the seed coat, the possibility of inducing biological genetic effects might be due to the radiation-induced bystander effect (RIBE). RIBEs are the phenomenon in which non-irradiated cells exhibit biological effects as a result of signals received from nearby irradiated cells (62). To test this hypothesis, the shoot apical meristem (SAM) and root apical meristem (RAM) of *Arabidopsis* seeds were shielded, and only the middle of each seed was irradiated. After 30 KeV ^40^Ar irradiation, various postembryonic development endpoints of SAM and RAM were inhibited (63). In another study, different parts of *Arabidopsis* R3L66 seeds (SAM-, RAM-, cotyledon-, and radicle-oriented) were irradiated, and significant increases in genetic changes (HR frequency and HR-related gene expression) were observed in the non-irradiated aerial parts of the irradiated plants (26). These results confirmed that long-distance bystander effects occur in plants. The temporal and spatial characteristics as well as the molecular mechanism of radiation bystander signals in plants have also been elucidated (64–67), and such mechanistic studies have provided strong evidence for clarifying the biological effects induced by low-energy particle irradiation. Currently, big data analysis technology is used to associate the radiation parameters of low-energy particles with the trait variation induced. It is expected that adjusting radiation parameters, such as the type of irradiated particles, dose or energy, will overcome the randomness of mutation and promote further development in the field of low-energy particle mutation breeding.

## Research On Space Breeding Of Plants

### Continuous Low-Dose and Combined Irradiation of Different Radiation Sources in a Space Environment May Be Important Factors Inducing Genetic Variation

Compared with on Earth, radiation and microgravity are two important factors that affect living organisms in space (68). Radiation is mainly produced by solar cosmic rays (SCRs) and galactic cosmic rays (GCRs). The radiation in low-Earth orbit also includes particles captured by the Earth's radiation belt, such as high-energy protons, heavy-ions, electrons, neutrons, and gamma rays (69). Among them, high atomic number and high energy particles in the heavy ion component, typically referred to as HZE particles, which are able to penetrate the spacecraft cabin and produce many secondary particles (70) in the spacecraft cabin, including gamma rays, electrons, protons, neutrons, and other heavy ions with different LET values. Long-term space flight test results show that the overall average absorbed dose rate in a low-Earth orbit spacecraft cabin is generally 0.1 to 0.5 mGy/d (71).

Although the space radiation dose rate and total absorbed dose are very low, the peak energy of HZE particles can reach 10_3_ MeV, and LET can reach more than 100 keV/μm, which has strong penetrability and ionization ability. Studies have shown that clustered DNA damage and DSBs induced by high-LET radiation are often difficult to accurately repair, especially in heterochromatin areas, and may even be irreparable (72). In addition, cells exhibit hyper-radiosensitivity (HRS) (73) and inverse dose rate effects (IDREs) (74). Therefore, long-term continuous exposure to low-dose composite radiation from different radiation sources in the space environment may produce considerable mutagenic effects. After short-term space flight, the mutation frequency of specific genes in yeast and *Caenorhabditis elegans* is 2 to 3 times higher than that of the ground control (75). The measurement results of rice seeds by the nuclear track radiation detection device carried by “Shenzhou 3” spaceship showed that 7 seeds directly bombarded by HZE particles introduced 10–15% molecular polymorphisms vs. contemporary control plants (76). Sun et al. also reported that space radiation induces epigenetic changes in plants and produces high-frequency mutations (77).

The microgravity of the space environment is another potential mutagenic factor. Anikeeva et al. found that microgravity can interfere with the DNA damage repair system, hinder or inhibit the repair of DNA damage, increase the sensitivity of plants to other mutagenic factors, and have a synergistic effect with radiation to aggravate biological mutations and increase the mutation rate (78). However, some studies have shown that the microgravity environment will not interfere with the biological effects of radiation (79). At present, it remains controversial whether there is a joint effect between radiation and microgravity (80).

### A Series of New Plant Varieties Derived From Space Mutation Have Been Released and Widely Applied in China

Many germplasm resources have been created using space breeding technology, and a large number of new plant varieties have been released in China. For example, in 1987, the Institute of Genetics of the Chinese Academy of Sciences cooperated with Guangxi Agricultural University to breed a new indica-japonica intersubspecific hybrid rice variety with strong hybridization, high seed-setting rate and full-filled grains (81). Xie et al. bred the restoration lines “Hang 1” and “Hang 2” by using space-based mutagenesis technology and developed a series of superhybrid rice varieties for large-scale production and application (82). Wang et al. obtained space-induced materials such as “Hanghui 1173”, “Hanghui 1179”, and highly rice blast-resistant “H4” and bred more than 50 rice varieties, including “Huahang 1” (83). In addition, stable and excellent varieties of wheat and sorghum developed by researchers from the Chinese Academy of Sciences and Chinese Academy of Agricultural Sciences have been obtained, such as wheat “Luyuan 502” (84). The Horticulture Branch of the Heilongjiang Academy of Agricultural Sciences and the Chinese Academy of Sciences have sent green pepper and tomato seeds on returnable satellites many times and selected high-yield, disease-resistant and good-quality space varieties “Yufan 1” and “Yufan 2” (85). The new space danshen variety “Tiandan No. 1” cultivated by the Tasly group has a single-plant quality three times that of ordinary danshen, and its active ingredient content is significantly higher than that of the control (86). Yuan et al. studied the variation frequency of mutated offspring derived from *Robinia pseudoacacia* seeds carried by the “Shijian 8” recoverable satellite and cultivated the new variety “hangci 4,” which showed a non-thorn trait (87).

## The Combination Of The Next-Generation Effective Particle Radiation And A High-Throughput Screening Method Will Further Improve The Efficiency Of Radiation Breeding

**Figure 4:**
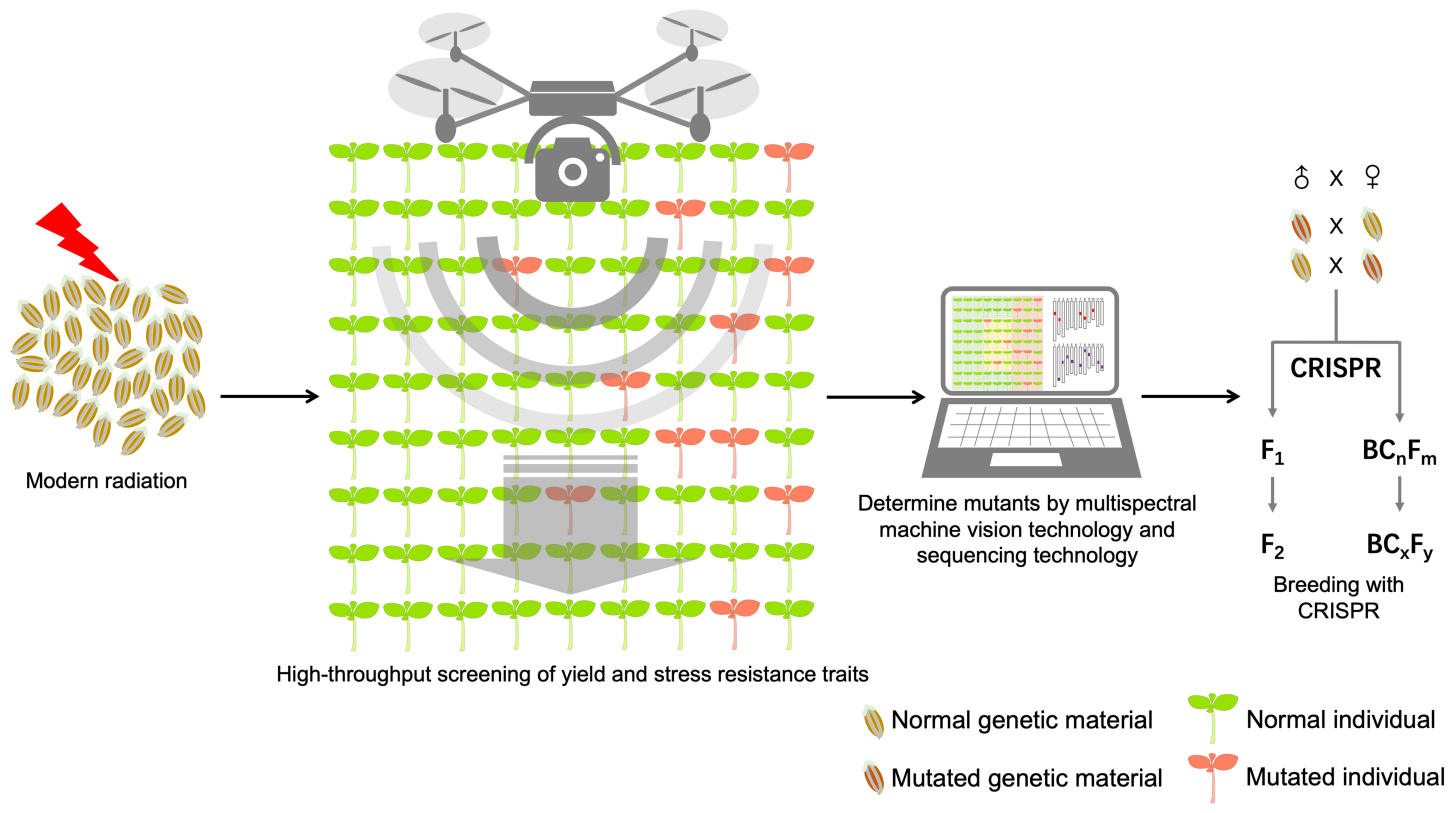
Next-generation effective modern particle radiation and high-throughput screening combined breeding system.

Direct or indirect DNA damage caused by ionizing radiation is the most important factor in the introduction of genetic variation. Therefore, constantly developing radiation sources with higher ionization capacity and then controlling the precise release of ionization energy at biogenetically active sites of organisms, such as the shoot apical meristem (SAM) cells of seed embryos (88), through physical parameter adjustment can induce high-density DNA damage at the genome-wide level and introduce more genetic variation. Modern particle radiation technology that can efficiently induce DNA damage is the basis for the future development of radiation breeding. In addition, single-cell radiation treatment can avoid the chimaerism phenomenon of multicellular tissue after radiation, so gamete cells are potential radiation objects. Furthermore, the identification and screening of genetic variation induced by radiation is key in breeding protocols. Identification methods of genetic variation include phenotypic identification (89), cytological identification (90), and molecular identification (91). In recent years, the development of modern high-throughput instruments and their combination with molecular labeling technology have resulted in a variety of efficient, accurate, and systematic breeding techniques, which can be used for high-throughput identification of genotypes and phenotypes of mutagenized populations for multiple consecutive generations. Multispectral machine vision technology and image processing technology improve the efficiency and dimension of phenotype identification and help breeders find potential mutations more quickly (92). The effective combination of the abovementioned technologies and drones will break the bottleneck of phenotype identification and realize high-throughput scanning of yield and stress resistance. For starch, protein, oil and other chemical materials highly related to crop quality, near-infrared technology can realize non-destructive identification at the single seed level and pre-planting screening of seed populations, so identification could be advanced by one genetic generation (93). The screening of specific genomic sequences is the key to mining elite alleles. The combination of a mixed sample strategy, high-throughput targeted sequencing and DNA labeling technology can significantly reduce the identification cost of targeted sequences and greatly improve the efficiency of DNA variation identification (94). Furthermore, the germplasm identified by phenotype and genotype should be closely combined with classical and modern biotechnology breeding procedures to improve the utilization efficiency of germplasm. The mutant germplasm identified can be directly cultivated into new varieties or used as important parental material to indirectly produce new varieties, improving mutagenesis breeding efficiency and offering new germplasm (95). Wang Ping proposed a method combining three factors: the mutagenesis materials as the core, molecular marker screening as an aid, and field identification as a supplement. Based on the above method, a series of new rice varieties were cultivated (96). How to efficiently pyramid and utilize multiple superior mutation sites is an important challenge. The rapid development of genome editing technology provides a new way to solve this problem (97). Breeders can obtain enhanced germplasms harboring multiple elite mutation sites by recombining mutation fragments or accurately replacement mutation sites through genome editing technology. We summarize the next-generation effective modern particle and high-throughput screening combined breeding system, as shown in Figure 4.

## Discussion

During the past 60 years, radiation mutation breeding together with other mutation breeding methods has been widely used to improve several crops and determine gene functions, even though it is facing a bottleneck in the development process, and the booming gene-editing technology has also brought great challenges. Nonetheless, with the increasing human population, decreasing in arable farmland area and deterioration of climate and the environment (drought, extreme temperature), there are strong requirements for stress-tolerant crop breeding. Under such circumstances, we should emphasize the mutual development and joint use of multiple breeding approaches to further shorten the crop breeding cycle and improve breeding efficiency (98). A large number of breeding traits are complex quantitative traits, and gene editing or molecular breeding techniques based on a few genes are not ideal for the improvement of quantitative traits. Therefore, it is necessary to strengthen research on mutation breeding. However, similar to other breeding techniques, radiation mutation breeding has limitations, such as beneficial mutant frequency being relatively low and the direction and nature of variation being difficult to predict. Indeed, producing more useful varieties and elucidating radiation mutagenesis mechanisms remain scientific problems worthy of attention. Overall, our understanding of the mutagenesis mechanism of radiation breeding is still insufficient, and in-depth research needs to be conducted, including regarding the origin of mutations in progeny plants. As indicated above, genomic instability also leads to mutations, constituting a double-edged sword for crop breeding: it can not only increase the variation rate of progeny but also result in the instability of mutant traits. Thus, the role of genomic instability in plant mutagenesis needs to be uncovered. Relying on the development of advanced radiation devices and combining high-throughput gene sequencing and other advanced molecular biotechnology, it is expected that the mutagenic effect of radiation might eventually be predictable, allowing research to develop toward directional mutagenesis.

The establishment of an accelerated particle radiation device provides a fundamental guarantee for the application of modern radiation mutation breeding, and its diversified parameter combinations might allow for directional mutagenesis in plant breeding. As a new generation of radiation mutagenesis sources, particle radiation represented by heavy-ions has further improved mutation frequency and the mutation spectrum compared with classical radiation mutagenesis, such as gamma rays and X-rays. This new generation breeding technology can generate more combinations of gene mutation sites, thereby breaking the linkage inheritance of traits, and it is expected that more mutants with excellent traits can be obtained, offering a breakthrough in the creation of new crop cultivars. Although particle radiation mutagenesis has been widely used in crop breeding and molecular genetic mechanism research, the mutagenesis mechanism is very complicated, and the mutagenesis effects of different radiation physical parameters of particles varied. Therefore, the key to the efficient creation of mutants is to select the appropriate types of accelerated particles and their radiation physical parameters. Several screening strategies for optimizing radiation parameters at the phenotypic and molecular levels have emerged (40, 99, 100). These studies provide valuable experience and new ideas for the formulation of optimal radiation conditions in the future particle radiation mutation breeding process. With the continuous development of sequencing technology, the combination of accelerated particle radiation with whole-genome resequencing, transcriptome sequencing, and other technologies can be employed to deeply explore changes in the genome and transcriptome levels in crops after accelerated particle irradiation and to further clarify the mutagenic mechanism of particle radiation. Furthermore, the further upgrade of particle radiation mutagenesis technology is of great significance to improve the efficiency of mutagenesis breeding.

Space radiation further expands the scope of radiation breeding, and the study of mutagenic effects in the space environment involves multiple disciplines, such as space biology, genetics, mutagenesis, and breeding. Although many studies have confirmed the mutagenic effects of the space environment and a series of varieties have been selected through space mutation, there is still a lack of research on the molecular characteristics, molecular spectrum and genetic mechanisms of mutations induced by the space environment. The following aspects are worthy of in-depth discussion: (1) analysis of the synergistic mutagenic effects of microgravity and space radiation; (2) single-factor analysis and ground simulation of space radiation mutagenic factors; (3) single-cell mutation mapping of space radiation-induced mutation and genetic network construction; and (4) high-efficiency identification of space-induced variation and development of rapid fixation technology. The development of single-cell sequencing, high-throughput sequencing, and high-throughput detection technologies provides favorable conditions for studying the effects of space environmental mutagenesis and accelerating the utilization of genetic variation at the whole-genome level.

## Author Contributions

LM, TW, and TG jointly drafted the manuscript. All authors contributed to the literature search and analysis, and reviewed and approved the final manuscript.

## Funding

This research was supported by the fund of innovation center of radiation application (No. KFZC2021010401).

## Conflict of Interest

The authors declare that the research was conducted in the absence of any commercial or financial relationships that could be construed as a potential conflict of interest.

## Publisher's Note

All claims expressed in this article are solely those of the authors and do not necessarily represent those of their affiliated organizations, or those of the publisher, the editors and the reviewers. Any product that may be evaluated in this article, or claim that may be made by its manufacturer, is not guaranteed or endorsed by the publisher.
